# Neurogenic Traditional Chinese Medicine as a Promising Strategy for the Treatment of Alzheimer’s Disease

**DOI:** 10.3390/ijms18020272

**Published:** 2017-01-28

**Authors:** Sravan Gopalkrishnashetty Sreenivasmurthy, Jing-Yi Liu, Ju-Xian Song, Chuan-Bin Yang, Sandeep Malampati, Zi-Ying Wang, Ying-Yu Huang, Min Li

**Affiliations:** 1 School of Chinese Medicine, Hong Kong Baptist University, Kowloon Tong, Hong Kong, China; sravangs@hkbu.edu.hk (S.G.S.); liujingyi@hkbu.edu.hk (J.-Y.L.); juxian.song@gmail.com (J.-X.S.); nkyangchb@gmail.com (C.-B.Y.); deepu.pharma08@gmail.com (S.M.); wangziying.12@163.com (Z.-Y.W.); wongwingyu@hotmail.com (Y.-Y.H.); 2 Mr. & Mrs. Ko Chi-Ming Centre for Parkinson’s Disease Research, Hong Kong Baptist University, Kowloon Tong, Hong Kong, China

**Keywords:** Alzheimer’s disease, neurogenesis, traditional Chinese medicine

## Abstract

Hippocampal neurogenesis plays a critical role in the formation of new neurons during learning and memory development. Attenuation of neurogenesis in the brain is one of the primary causes of dementia in Alzheimer’s disease (AD), and, conversely, modulating the process of hippocampal neurogenesis benefit patients with AD. Traditional Chinese medicine (TCM), particularly herbal medicine, has been in use for thousands of years in Asia and many regions of the world for the treatment of cancer, cardiovascular diseases and neurodegenerative diseases. In this review, we summarize the role of neurotrophic factors, signal transducing factors, epigenetic modulators and neurotransmitters in neurogenesis, and we also discuss the functions of several Chinese herbs and their active molecules in activating multiple pathways involved in neurogenesis. TCM herbs target pathways such as Notch, Wnt, Sonic Hedgehog and receptor tyrosine kinase pathway, leading to activation of a signaling cascade that ultimately enhances the transcription of several important genes necessary for neurogenesis. Given these pathway activating effects, the use of TCM herbs could be an effective therapeutic strategy for the treatment of AD.

## 1. Introduction to Neurogenesis

Neurogenesis is defined as generation and development of new neurons, has become one of the most influential research areas in treating many neurodegenerative disorders such as Alzheimer’s disease, Parkinson’s disease, and Huntington’s disease. The central nervous system is intricately designed to transmit signals throughout the body, however, in the brains of Alzheimer’s disease (AD) patients, the connections between neurons are disrupted due to neurodegeneration. It was traditionally believed cell division of neurons is halted when neuronal cells are terminally differentiated. However, Reynolds and Weiss’s work in 1992 with isolated neural stem cells from adult mouse striatum challenged the theory of non-renewability of neurons. Nonetheless, neurogenesis in the brain is generally limited to a particular stage of brain development. Two exceptions to this rule are the dentate gyrus (DG) of the hippocampus and the sub-ventricular zone (SVZ) of the lateral ventricles, where actively dividing neurons are observed in all stages of brain development. In the SVZ, neural stem cells (NSCs) give rise to neuroblasts, which migrate toward the olfactory bulb (OB) and differentiate into inter-neurons. In the DG, granule cells (GCs) extend their dendrites into the GC layer and their axons into the hilus toward the Cornu Ammonis 3 (CA3) region, giving rise to mature and integrated GC [1,2]. Newly generated neurons in the adult brain makes several connections and receive several inputs from pre-existing mature neurons [3]. During migration, differentiation and maturation of progenitor cells, there is a balance between survival and apoptosis of the neurons that play key roles in determining the neuronal cell number and their function in the adult brain [4]. Furthermore, each of these stages is dysregulated during pathological disease conditions. Each of the many facets of neurogenesis—the many pathways, regulatory mechanisms and molecules—could be a subject for research, for reversing degeneration and restoring neuronal generation.

## 2. Regulatory Mechanisms of Neurogenesis

With the advent of molecular techniques, through in vivo, in vitro and in silico experiments, scientists have delineated signaling pathways involved in neurogenesis. Stem and progenitor cells are highly controlled by several extrinsic and intrinsic factors. Extrinsically these cells are regulated by molecular determinants including Wnt/β-catenin, the Sonic Hedgehog (Shh) and Notch pathways, neurotransmitters, and neurotrophins, all of which are abundantly present in the SVZ and SGZ. Furthermore, cell-intrinsic mechanisms comprising epigenetic regulators and transcription factors have been shown to be critical modulators of neurogenesis in the adult brain.

### 2.1. Neurotrophic Factors

Neurotrophic factors are soluble proteins, specific to cell surface receptors. Upon binding to the receptors, they exert diverse effects, ranging from proliferation to maturation of cells and also provide trophic support. There are four mammalian neurotrophic factors, i.e., nerve growth factor (NGF), brain-derived neurotrophic factor (BDNF), neurotrophin-3 (NT-3) and neurotrophin-4/5 (NT-4/5), all of which have been studied extensively for their neuronal survival activities. Neurotrophic factors target and activate one or more Trk family of receptor tyrosine kinases and p75 receptors. NGF specifically activates TrkA, BDNF and NT-4/5 activates TrkB, and NT-3 is specific to TrkC [5].

Amongst the other genes involved in neuronal functions, members of the neurotrophin family of neurotrophic factors (NTFs) play essential roles in synaptic transmission and neural development [6,7]. In addition, a strong link has been established between BDNF expression and neurogenesis [8,9]. Studies have shown the importance of BDNF signaling pathways in the regulation of neurogenesis in the parietal cortex, entorhinal cortex, frontal lobe, temporal cortex and the hippocampus. mRNA transcript levels of BDNF are also severely affected during neurodegenerative diseases such as Alzheimer’s disease [10,11].

Nerve growth factor (NGF) also has roles in augmenting new hippocampal neuron survival, probably by increasing hippocampal cholinergic activity [12]. Some evidence from knockout and knockin animal models show neurotrophin-4 (NT-4) and neurotrophin-3 (NT-3) promote hippocampal neurogenesis, and improve spatial memory and long-term potentiation (LTP) [13,14,15].

### 2.2. Signal Transduction

#### 2.2.1. Sonic Hedgehog Pathway

**Figure 1:**
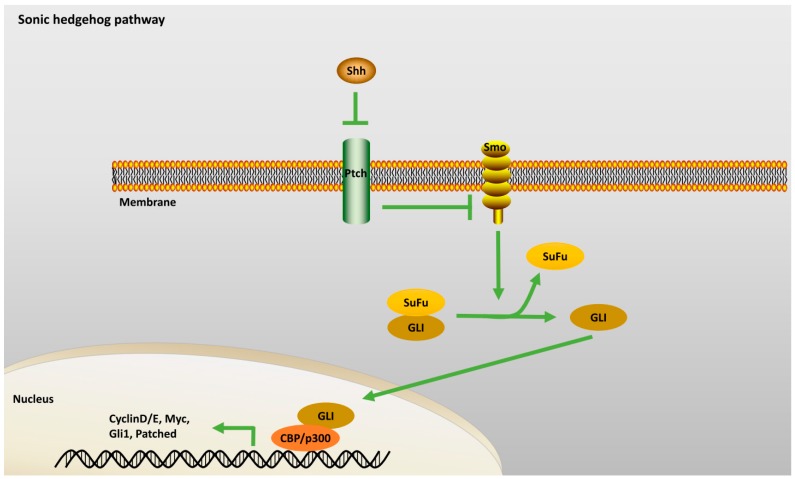
Sonic Hedgehog Pathway: Shh acts on Ptch to inhibit the repression of Smo by Ptch. GLI releases from SuFu to promote transcription of target genes upon binding to CBP/p300. Green arrow represents “activation”. Abbreviation: Shh: Sonic Hedgehog; Ptch: Patched; Smo: Smoothened; CBP: CREB binding protein; SuFu: Suppressor of Fused.

Shh is a soluble extracellular signaling protein which is vitally involved in neural tube generation and differentiation. Shh exerts its action via the signaling receptor complexes (Figure 1) that are composed of transmembrane receptor protein Patched (Ptch) and G-protein coupled co-receptor Smoothened (Smo). When Smo is in an active state, interaction of GLI with its suppressor complex (SuFu) is blocked. Thus, activated GLI promotes transcription of its target genes. Shh plays a significant role in adult rat hippocampal progenitor stem cell proliferation in vitro and in vivo. Immunohistochemical analysis and in situ hybridization data show a consistent presence of Ptch and Smo in the DG regions of adult rats. It was also shown that recombinant Shh induces multipotency of progenitor cells giving rise to neurons, glial cells as well as oligodendrocytes [16].

#### 2.2.2. Wnt Pathway

**Figure 2:**
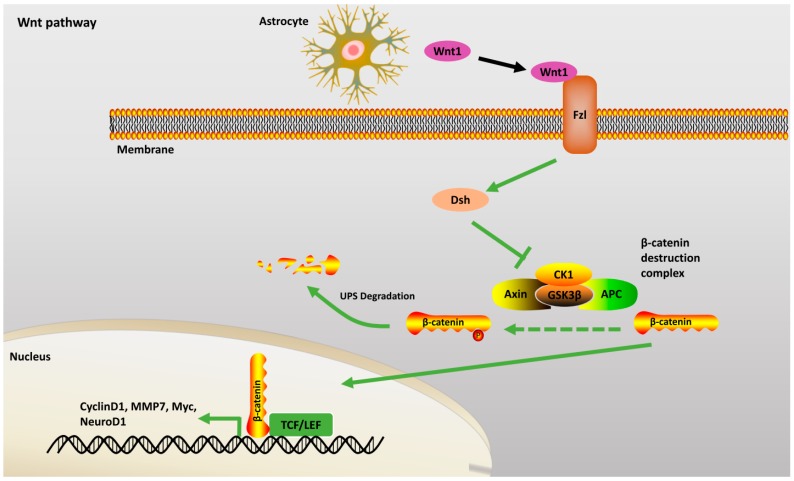
Wnt Pathway: In the presence of Wnt ligands, Dsh blocks the β-catenin destruction complex consisting of Axin, APC, CK1, GSK3β. β-catenin translocates into the nucleus and activates TCF/LEF, that can activate its target genes. Green dashed arrow: inhibitory. Abbreviations: APC: Adenomatous polyposis coli; GSK3β: Glycogen synthase kinase 3β; Dsh: Dishevelled, UPS: ubiquitin proteasome system; MMP7: matrix metalloproteinases; CK1: Casein kinase1; TCF/LEF: T cell factor/lymphoid enhancer-binding factor; Fzl: Frizzled.

Wnt proteins are produced in the hippocampal astrocytes which govern the differentiation and proliferation of neural progenitor cells (NPCs) [17,18]. During embryogenesis, a coordinated interaction between multiple interacting partners such as β-catenin, adenomatous polyposis coli (APC), GSK-3β, Axin and Conductin is observed [19]. For successful Wnt signaling to take place, Wnt proteins interact with GSK3β and inhibit its action, thereby preventing phosphorylation of β-catenin and consequently rescuing it from UPS degradation. The buildup of cytoplasmic β-catenin translocates into the nucleus. It interacts with lymphocyte enhancing factors (LEFs) and T-cell factors (TCFs) to activate transcription of multiple target genes (Figure 2). By the orchestration of several transcriptional factors, Wnt signaling promotes the proliferation of early NPCs [20]. Many studies have shown that this signaling through Wnt stimulates neuronal and astroglial differentiation but inhibits oligodendroglial differentiation [21].

#### 2.2.3. Notch Pathway

**Figure 3:**
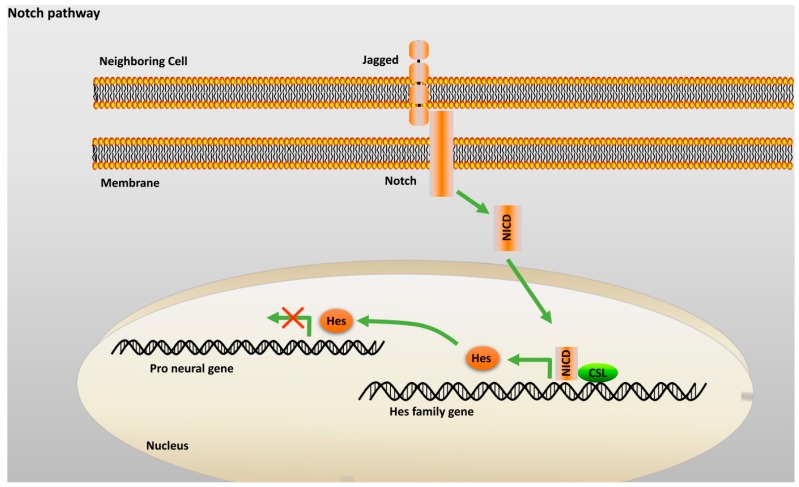
Notch Pathway: Interaction of Notch with Jagged on the neighboring cell, leads to the release of NICD that translocate into the nucleus and binds to CSL thereby aiding in transcription of Hes family of repressor proteins. Hes functions by blocking the transcription of pro neural genes. Green arrow: activation. Red cross: inhibition of transcription. Abbreviation: NICD: notch intracellular domain; CSL: CBF1, Suppressor of Hairless, Lag-1.

Notch is a single pass transmembrane protein largely involved in determining the fates and lineage of developing neural stem cells (NSCs) [22,23]. Notch transmembrane receptors are activated by Delta and Jagged, two other single pass transmembrane proteins, after which the intracellular portion of the membrane is cleaved by γ-secretase to release Notch intracellular domain (NICD) [24]. The released NICD translocates into the nucleus and complexes with Centromere binding factor-1 (CBF1), a transcription regulator (Figure 3). Several studies have shown that this complex activates target genes, such as Hes, Hes5 and ErbB2, which, when transcribed, aid in the proliferation and maintenance of stem cells and in inhibiting neural differentiation [25,26].

#### 2.2.4. Receptor Tyrosine Kinase Pathway

**Figure 4:**
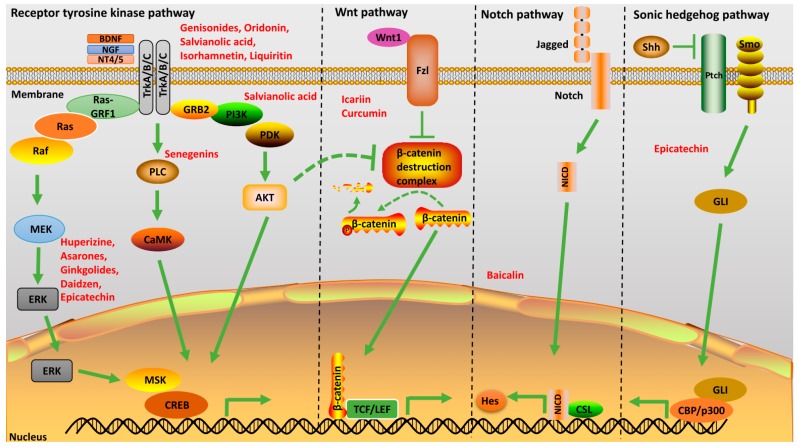
Schematic description of neurogenesis of traditional Chinese medicine (TCM). TCM active ingredients can activate neurogenic pathways such as Wnt, Notch, Shh and Neurotrophic factors. Activation of neurotrophic factors by TCMs can activate three major pathways downstream of Trk receptors, namely MAPK/ERK, PLC and PDK. The active effector molecules translocate into the nucleus and activate genes necessary for neurogenesis. Dashed line and dashed arrow: inhibition. Green arrow: activation. Abbreviation: MAPK: mitogen-activated protein kinase; MEK: MAPK/ERK Kinase; ERK: Extracellular signal-regulated kinase; CREB: cAMP response element-binding protein; MSK: Mitogen and stress activated kinase; PKC: Protein kinase C; PI3K: phosphotidyl inositol-3 kinase; RasGRF1: Ras Protein Specific Guanine Nucleotide Releasing Factor 1.

Post-translational modifications such as phosphorylation are key modifications observed in several proteins involved in propagating extracellular cues for intracellular signal transduction. Hence, ligand binding to the receptor tyrosine kinases (RTKs) results in phosphoryl activation/inhibition of several downstream targets. RTKs orchestrate the activation of several pathways associated with several cellular phenotypic responses (Figure 4). These include signaling cascades mediated by Ras/Raf/MAP kinase, phosphoinositide-3-kinase (PI3K)/Akt and phospholipase Cγ (PLC-γ) [27,28].

Tropomyosin-related kinase receptors (TrkA, TrkB and TrkC) play an essential role in the development and maintenance of central nervous system (CNS) and peripheral nervous system (PNS), in the reversing of neurodegeneration and in the augmentation of synaptic plasticity [5,29,30].

### 2.3. Miscellaneous Regulators of Neurogenesis

#### 2.3.1. Roles of Epigenetic Modulators in Neurogenesis

Epigenetics is the study of inheritable changes in cellular phenotype without any change in the DNA sequence. It involves post-translational modification on N-terminal tails of histones. Several chromatin modulators, namely histone methyltransferases (HMTs), histone demethylases (HDMs), histone acetyl transferases (HATs), histone deacetylases (HDACs), and chromatin remodelers, play crucial role in gene regulation processes such as DNA replication, transcription and repair. In proliferative neural stem cells and post-mitotic newborn neurons, epigenetic modulators produce lasting memory and hereditary effects that orchestrate conversion of one cellular state to another during neurogenesis. These mechanisms serve in fine-tuning and regulating the expression of several genes necessary during neurogenesis [31].

#### 2.3.2. Roles of Neurotransmitters in Neurogenesis

Neurotransmitters were initially thought to simply mediate transfer of signals from one cell to another. Based on several studies, it was later shown that neurotransmitters also have functional roles in neurogenesis in normal and pathological states. Neurotransmitters are generally categorized as amino acids (GABA and glutamate), cations (acetylcholine), monoamines (serotonin, dopamine and noradrenaline), neuroactive peptide (neuropeptide Y) and soluble gases (nitric acid) [32]. When neurotransmitters are modulated, they can influence neurogenesis to produce functional neurons. The actions of these neurotransmitters are context-dependent and diverse in nature [33,34,35]. Several of the neurotransmitters can activate the production of trophic factors such as BDNF. For example, stress-induced down-regulation of BDNF mRNA can be reversed by treatment with serotonin reuptake inhibitors (SSRIs) [36], hence showing an interaction between neurotransmitters and neurogenesis.

## 3. Neurogenesis and Alzheimer’s Disease

Alois Alzheimer was the first physician to observe signs of AD, i.e., senile plaques and neurofibrillary tangles (NFTs), the physiological changes now recognized as hallmarks of the disease [37]. In AD, the early stages of neural degeneration appear in the transentorhinal cortex and extend to the entorhinal cortex and hippocampus. Later, the degeneration spreads towards the temporal, frontal and parietal lobes, leading to the loss of neurons and synaptic contacts [38]. Impairment in neurogenesis is very relevant to AD progression as the disease affects the two neurogenic niches in the brain [39,40].

Ample evidence shows that AD and impaired neurogenesis share numerous common molecules and pathway circuits involved in pathological states of newborn neurons [41,42,43]. It has been shown that a differential regulation of neurogenesis in the subgranular and the subventricular zones in nine-month-old APPswe/PS1dE9 transgenic mice. Although the number of PCNA-positive cells in both the neurogenic zones were normal, a decreased number of BrdU-positive cells was observed in the subgranular zone, as compared to normal number of BrdU-positive cells in the subventricular zone [44]. In one of the studies there were reductions in the numbers of BrdU-positive cells and of neuroblasts (DCX-positive cells) in nine-month-old APP Swedish PS1 dE9 animals, leading to impairment in neurogenesis [45]. Furthermore, six-month-old APPxPS1-Ki transgenic mice with brain amyloidosis along with neuronal loss, displayed a significant reduction in the hippocampal pyramidal cell layer, along with reduced neuronal bulk in the dentate gyrus, demonstrating a causal link between AD and impaired neurogenesis [46].

## 4. Neurogenic Potential of Traditional Chinese Medicine (TCM) Herbs and Its Active Ingredients

Tremendous efforts have been devoted to the development of drugs to treat AD. To date, five drugs known as the acetylcholinesterase inhibitors and *N*-methyl-d-aspartate receptor antagonist have been approved by the U.S. Food and Drug Administration (FDA) in the treatment of AD. However, they can only provide symptomatic relief of learning and memory dysfunction without curing the disease. Moreover, the prolonged use of these drugs may cause severe adverse effects, such as hepatotoxicity and cholinergic crisis [47]. Due to the limitation of the available medications, researchers are looking for new therapies with breakthrough, and drugs that can stop the cell damage and eventual neuron loss and promote neuron regeneration that are of great value [48].

TCM therapies are advantageous for treating complicated diseases due to their nature of multiple components that target multiple signaling pathways simultaneously, thereby benefiting the body as a whole. In recent years, the protective effect of TCMs and their bioactive ingredients on adult neurogenesis in both in vivo and in vitro experimental AD models attracts increasing attention [49]. Meanwhile, TCMs are also effective on the oxidative stress and inflammation, which are closely associated with AD pathology. From this perspective, TCMs and their bioactive ingredients provide a valuable database for drug screening and development in the treatment of AD. Here, we summarize ten commonly used TCMs with prominent neurogenic effects (Table 1), and in subsequent text, describe five of them in detail.

### 4.1. Curcuma longa

*Curcuma longa* is a food additive, coloring agent and spice widely used in Indian culinary preparations [60]. Curcumin (diferulomethane) is the primary active compound; it has anti-oxidative, anti-inflammatory, chemo-preventative properties [61,62,63] with a favorable toxicity profile [64,65]. According to traditional Indian medicine, curcumin is considered to have several healing properties for many pathological conditions, ranging from asthma to epilepsy, from kidney stones to cancer [66].

Recent studies have shown that curcumin has a role in enhancing hippocampal cellular proliferation and improving cognitive abilities in aged mice. Comparison of 6- and 12-week-old Sprague-Dawley (SD) rats showed an increase in numerous neural development genes involved in neurogenesis. When curcumin was feed-administered to male Sprague-Dawley (SD) rats for six or 12 weeks, a time-dependent improvement in spatial memory and better cognition was observed, shown by Morris water maze, open field, Rota-rod and social recognition. Exon array assay showed an increase in neurogenesis-related genes modulated in both cortex and the hippocampus [67]. In another study, chronic administration of curcumin in stressed rats increased neurogenesis, via increasing the protein and mRNA levels of BDNF [68]. Chronic (21 days) curcumin treatment upregulates p-CREB and BDNF levels, which protects from chronic stress-induced damage to hippocampal and frontal cortical neurons [69]. Hence, curcumin is a potent activator of BDNF-CREB pathway which is well-known to be involved in neurogenesis.

### 4.2. Huperzia serrata

*Huperzia serrata* is a club moss that has been used in China and across the world for treating medical ailments such as swelling, bruises, and fever as well as disorders such as myasthenia gravis, schizophrenia and blood disorders. Huperzine A (Hup A) is one of its major constituents and an active ingredient. Hup A is a sesquiterpene alkaloid, which is a potent reversible inhibitor of acetylcholinesterase (AChE) [70,71,72,73]. Hup A has been extensively studied by many research groups in China, for the treatment of age-related memory disorders including AD. Treatment with Hup A significantly increases neuritic outgrowth in cultured undifferentiated PC12 cells and also enhances the secretion of NGFs in rat cortical astrocytes [74]. Research has also shown that Hup A robustly improves Mini-Mental State Examination (MMSE) and activities of daily living (ADL) scores in patients suffering from AD [75]. Pretreatment of human neuroblastoma cells SH-SY5Y with 10 µM Hup A protects the cells from H_2_O_2_ induced cytotoxicity possibly by restoring the NGF to normal levels via the activation of downstream MAP/ERK/CREB signaling cascade [76]. Altogether, Hup A is considered as a well-tolerated drug for AD with potential neurogenic activity in vitro and in vivo.

### 4.3. Panax ginseng

*Panax ginseng* is one of the most popular traditional herb, used in treating many ailments for over 2000 years in China, India, Korea, Japan and now, worldwide. Ginseng has been rigorously studied and researched for its antioxidative activity, anti-ageing, antidepression, anti-anxiety properties and anti-cognitive impairment induced by neurological disorders [77]. Three *Panax* species have been extensively investigated: (1) *P. ginseng*; (2) *P. quinquefolium* L; and (3) *P. notoginseng* [78].

Ginsenosides are the most active ingredients in *Panax ginseng* and its beneficial effects have been well-documented. The activity of ginsenosides ranges from nourishment to anti-cancer activity [79]. Based on structural differences, ginsenosides are classified into three main categories, namely panaxadiols, panaxatriols and oleanolic acid groups. The panaxadiol group includes Rb1, Rb2, Rb3, Rc, Rd, Rg3, Rh2, and Rs1, whereas the panaxatriol group includes Re, Rf, Rg1, Rg2, and Rh1. Ro belongs to the oleanolic acid group [80]. Among the other beneficial effects of ginsenosides, Rg1 possess neurogenic properties as it enhances neural proliferation in adult male Kunming mice. It has also been shown to increase cell proliferation in cultured primary neurospheres and enhance the survival rate significantly in hippocampal progenitor cells [81]. It has been demonstrated that Rg1 increases neuronal progenitor cell number through the activation of the cAMP–CREB signaling pathway which is very well known for activating genes related to neurogenesis [82].

### 4.4. Salvia miltiorrhiza

*Salvia miltiorrhiza* has been used for the treatment of cerebrovascular and cardiovascular diseases [83,84], whose bioactive ingredients are liposoluble diterpenoid and hydrosoluble phenolic acids. Among them, liposoluble diterpenoids include tanshinone I, tanshinone IIA, tanshinone IIB, cryptotanshinone and dihydrotanshinone I; the hydrosoluble phenolic acids present include Salvianolic acid A, Salvianolic acid B and danshensu. Salvianolic acid B (Sal B) is the most abundant and a major active ingredient in the water extract. Traditionally, Sal B was used to treat cerebrovascular, cardiovascular and neurodegenerative disorders [85,86,87]. Mice receiving Sal B (25 mg/kg) intracerebroventricularly showed significantly improved spatial learning and also memory improvement and also increased the expression levels of anti-inflammatory cytokines IL-10 and TGFβ, suppressed the pro-inflammatory cytokines IL-1β and TNFα [88] and also decreased the inflammatory astrocytes and microglial cells [54,89]. Furthermore, Sal B promotes cell growth and differentiation by activating ERK and PI3K/AKT pathways [90]. Recent studies have shown SalB time- and dose-dependently increased the activation of neural stem cells in rat embryonic cortex, suggesting that Sal B is a potential candidate that can promote neurogenesis and thus benefit patients with AD [91].

### 4.5. Ginkgo biloba

Historically, the ginkgo tree has been considered to have a lot of medicinal value and hence has been used to treat clinical symptoms of aging, peripheral vascular disorders and many neurological symptoms similar to those of AD. In the early 1970s, Willmar Schwabe Pharmaceuticals (Karlsruhe, Germany) standardized the extraction procedures of stable extracts (EGb761) from *Ginkgo* leaves [92]. The extract of *G. biloba* leaves contains 24% flavonoid glycosides, making them the major constituent of the extract, followed by 6% terpenoids and 5%–10% organic acids. Terpenoids and flavonoids together form the most active components of the leaf extract (EGb761). The major flavonoids in ginkgo are quercetin, kaempferol, and isorhamnetin; the terpenoids are ginkgolides A, B, C and bilobalide [93].

Transgenic AD mice (TgAPP/PS1 and Tg2576) receiving extracts of EGb761 showed increased hippocampal activity. Induction of neurogenesis was associated with the activation of CREB, which reduced the formation of oligomeric Aβ in the AD mice [94,95,96]. Treatment of mice with bilobalide and quercetin, two natural active constituents in ginkgo, showed an increase in and maintenance of newly formed dendritic spines. This treatment also promoted neurogenesis by increasing the expression of p-CREB and BDNF [97,98,99]. All of these studies demonstrate the efficacy and therapeutic potential of *Ginkgo* in the treatment of age- related neurodegenerative damages.

## 5. Miscellaneous TCM Active Molecules Showing Neurogenic Potential

Isorhamnetin, one of the major active flavonol aglycones isolated from *G. biloba*, has been shown to induce the expression of neurofilaments, components of neuronal axons, and also potentiate the neurite-inducing activity of NGF [100]. Isorhamnetin and other flavonols, such as kaempferol and quercetin, are also involved in increasing the expression of synaptophysin, synapsin and GAP-43, which are the neuronal differentiation markers [101] suggesting flavonols may play significant roles in neurogenesis.

Liquiritin, a major flavonoid found in *Glycyrrhizae radix*, has been used in the Traditional Chinese medicinal system for the treatment of asthma, peptic ulcers, anti-cancer and anti-HIV therapies. Recent studies have shown that liquiritin is beneficial in the treatment of neurodegenerative diseases such as AD [102]. Treating cells with liquiritin along with low doses of NGF aids in the enhancement of neurite outgrowth probably by increasing the expression levels of important neuronal related genes such as Neurog 3, Nf 1, Notch 2, Nmur 2 and Ntf 5, ultimately leading to neural regeneration, differentiation and synapse formation [103].

Daidzein, a dihydroxyflavone present in the tubers of *Radix Puerariae* which has been used to treat cold, fever, bronchitis, and cough [104]. It is known to show neurogenic activity in hippocampal neurons, cortical neurons, Dorsal Root Ganglion (DRG) neurons, and PC12 cells [105,106,107]. It promotes neurogenesis by stimulating the Src-ERK pathway that can activate several downstream neurogenesis-related genes [108].

Astragaloside IV (AS-IV) is the active ingredient extracted from the dried root of *Astragalus membranaceus (A. membranaceus)*. *A. membranaceus* is well-known in TCM for its anti-inflammatory, anti-oxidative, cardio-protective and immune system-stimulating activities. It has been demonstrated that methanolic extracts of *A. mongholicus* reverse the effect of Aβ (25–35)-induced neuritic atrophy and memory loss in the hippocampus and cerebral cortex of mice. The individual active ingredients, astragaloside I, astragaloside II, and astragaloside IV, promote axonal extension and cell survival [109].

Asarones are important active ingredients of *Acori tatarinowii* (AT). Asarones has been shown to positively regulate the proliferation of neural progenitor cells and improve hippocampal neurogenesis in C57BL/6 mice via increasing the phosphorylation and activated forms of ERK, without showing any change in AKT signaling pathway. Activation of ERK and its downstream signaling molecules have been shown to be a prerequisite for neural progenitor cell differentiation and hence neurogenesis [55].

Epicatechin has been found to be the most abundant and the most active polyphenol present in grape seed extracts [110] and cocoa beans [111]. Significant improvement in memory was observed in 12-month-old female Tg2576 AD transgenic mice when administered with a grape-derived polyphenolic preparation (GP). In addition, significant improvement in memory retention was observed in mice that received treatment compared to the control. Moreover, the bioavailability of methylated glucuronide metabolites of epicatechin was increased in the brain, concentrations reaching >300 pmol/g. Furthermore, this increase aided in maintaining LTP and synaptic transmission, and in improving learning and memory, as demonstrated by spatial behavioral assessment. It was also observed that the possible mechanism is via activation of CREB which is necessary for memory and learning [112,113].

Oridonin, a diterpenoid, is one of the important active ingredients from the Chinese herb *Rabdosia rubescens*. It is well-known for its anti-cancer [114] and anti-inflammatory [115] functions. Study has demonstrated that Aβ-induced neurotoxicity and synaptic dysfunction was alleviated with the administration of oridonin in AD mice. Oridonin shows significant neuro-protective effects by activating the BDNF/TrkB/CREB signaling pathway, which is often activated during the process of neurogenesis [57].

Kami-ondam-tang (KOT; Jiawei-wen-dan-tang in Chinese; Kami-untan-to in Japanese) is an herbal formulation that has been prescribed for the alleviation of neurological problems such as neurosis and insomnia [116,117]. Sub-chronic administration of KOT in male Institute of Cancer Research (ICR) mice improved spatial memory and cognitive behavior via activation of memory markers such as p-CREB, p-AKT, BDNF and Nestin [118]. Another Chinese herbal formulation, Fuzhisan (FZS), also promotes neurogenesis. It is found in the roots of *Panax ginseng*, Baical skullcap, and rhizomes of *Acorus calamus* L and *Radix glycyrrhizae*. Intra-gastric administration of FZS in mouse improved cognitive behavior and promoted proliferation and differentiation of neural stem cells [119]. Danggui-Shaoyao-San (DSS) is a well-known herbal formulation which has been used for centuries in China, Japan and Korea to treat gynecological disorders [120]; it also acts as an anti-depressant [121] and possesses anti-apoptotic affects [122]. Evidence also suggests that DSS has significant neuroprotective effects [123] by reversing cognitive deterioration and maintaining the cortical structure in the brains of aged mice. Immunohistochemistry of DSS-treated SD rats showed a significant increase in the number of BrdU+/DCX+ cells in the SVZ as compared to the control group [124].Based on all of the above studies, in general, it appears that traditional Chinese medicinal compounds exert neurogenic activity by regulating downstream signaling pathways that potentiates expression of genes necessary for neuronal outgrowth and neurogenesis.

## 6. Conclusions

Neurogenesis is a vital process for maintaining the structural integrity of neurons for cognitive behavior and memory, especially in the adult brain. This process can be regulated through neurotrophic factors, neurotransmitters, epigenetic factors and signal transduction including Wnt, Shh and RTK pathways. Under pathological conditions, the disruption of those regulatory mechanisms may lead to neuron loss and eventually cause neurodegenerative diseases, such as AD. Through the ages, TCM herbs have shown good efficacy in treating many and complex diseases due to the synergistic effects of their multiple components. In the treatment of AD, successful examples are *Curcuma longa*, *Panax ginseng*, *Ginkgo biloba*, and *Salvia miltiorrhiza*. Their bioactive components and underlying mechanisms involving the regulation of neurogenesis have been summarized in this review. In conclusion, TCMs have great neurogenic potential and represent promising therapeutic agents for AD treatment.

## Figures and Tables

**Table 1:** Summary of selected Traditional Chinese medicine (TCM) showing neurogenic activity in vitro and in vivo.

No.	Active Ingredients	Herb Source	Class	Modes of Delivery In Vivo/In Vitro	Assay (In Vivo/In Vitro)	Observations	Possible Mechanisms	Reference
1	Curcumin	*Curcuma longa*	Polyphenol	Intraperitoneal	In vivo: Conditioned Avoidance test In vitro: Neurosphere assay, Luciferase reporter assay	Increased p-GSK-3β, Reelin and Pax6 in the hippocampus	Canonical Wnt/β-catenin pathway	[50]
2	Huperzine A	*Huperzia serrata*	Alkaloid	Intraperitoneal	In vitro: CCK-8 cell proliferation assay	Neural stem cell proliferation	MEK pathway	[51]
3	Ginsenosides	*Panax ginseng*	Glycoside	Intraperitoneal	In vivo: BrdU administration In vitro: VEGF and BDNF assay	Increased VEGF and BDNF	AKT and ERK pathway	[52]
4	Ginkgolide	*Ginkgo biloba*	Prenol	Oral	In vivo: BrdU administration In vitro: Immunoblotting	Inhibition of Aβ oligomerization	BDNF/CREB pathway	[53]
5	Salvianolic acid B	*Salvia miltiorrhiza*	Phenolic acid	In vitro cell culture	In vitro: Neurosphere culture, RT-PCR, Immunofluorescence	Decreased LDH leakage, inhibition of apoptosis	BDNF activation	[54]
6	Asarone	*Acori tatarinowii*	Phenyl proponoid	Intraperitoneal	In vivo: BrdU administration In vitro: Immunoblotting	NPC proliferation, Neurogenesis	MEK pathway	[55]
7	Epicatechin	*Camellia sinensis*	Flavanoid	Intraperitonieal	In vivo: BrdU administration In vitro: Immunoblotting	Elevated BDNF in hippocampus	Increase Tyrosine hydroxylase levels in brain	[56]
8	Oridonin	*Rabdosia rubescens*	Terpenoid	Intraperitonieal	In vivo: Morris water maze. In vitro: Synaptosomal activity assay, Golgi staining	Increase in PSD95, Synaptophysin, BDNF, p-TrkB and p-CREB	BDNF/TrkB/CREB pathway	[57]
9	Catalpol	*Rehmannia glutinosa*	Iridoid	Oral	In vivo: Y-maze. In vitro: AChE activity, Radio-ligand binding assay, ChAT activity	Increase in M-receptor density and neuritic outgrowth	BDNF/TrkB/CREB pathway	[58]
10	Senegenin	*Polygala tenuifolia*	Iridoid	In vitro cell culture	In vitro: PC12 neurite outgrowth experiment	Neuronal outgrowth, Increase in GAP-43 and MAP2	PKC pathway	[59]

## Abbreviations

AchE	Acetylcholine esterase
AD	Alzheimer’s Disease
BDNF	Brain-derived neurotrophic factor
CREB	Cyclic adenosine 3,5-monophosphate responsive element binding protein
DG	Dentate gyrus
GAP43	Growth associated protein 43
GC	Granule cell
GSK3β	Glycogen synthase kinase 3β
LDH	Lactate dehydrogenase
MAP2	Microtubule associated protein 2
MAP	Mitogen activated protein kinase
NFT	Neurofibrillary tangles
NGF	Nerve growth hormone
NICD	Notch Intracellular domain
NSC	Neural stem cell
NT	Neurotrophin
OB	Olfactory bulb
Ptch	Patched
RTK	Receptor tyrosine kinase
Shh	Sonic hedgehog
Smo	Smoothened
SVZ	Sub-Ventricular zone
Trk	Tropomyosin-related kinase
TCM	Traditional Chinese medicine
